# Musculoskeletal diseases, infections and vaccines: state of the art, research perspectives and educational needs

**DOI:** 10.1007/s40520-025-02940-w

**Published:** 2025-02-22

**Authors:** Fiona Ecarnot, Jotheeswaran Amuthavalli Thiyagarajan, Mario Barbagallo, Jane Barratt, Stefan Constantinescu, Ori Elkayam, Luigi Ferrucci, Mickaël Hiligsmann, Meliha Kapetanovic, Francesco Macchia, Jean-Pierre Michel, Alberto Migliore, Alberto Pilotto, Cornel Sieber, Anja Strangfeld, Nicola Veronese, Davide Liborio Vetrano, Stefania Maggi, René Rizzoli

**Affiliations:** 1 SINERGIES, University of Franche-Comté, Besançon, 25000 France; 2 Department of Cardiology, University Hospital Besançon, Besançon, 25000 France; 3 Department of Maternal, Responsible Officer for Bone Health and Ageing Initiative, Ageing and Health Unit, Newborn, Child, Adolescent Health and Ageing, World Health Organization, Geneva, Switzerland; 4 Department of Internal Medicine and Geriatrics, Geriatric Unit, University of Palermo, Via del Vespro 141, Palermo, 90127 Italy; 5 International Federation on Ageing, Toronto, Canada; 6 Federation of European Academies of Medicine, Brussels, Belgium; 7 Department of Rheumatology, Tel Aviv Medical Center, Tel Aviv Medical Center and the “Sackler” Faculty of Medicine, Tel Aviv, Israel; 8 Intramural Research Program of the National Institute on Aging, Baltimore, MD USA; 9 Department of Health Services Research, CAPHRI Care and Public Health Research Institute, Maastricht University, Maastricht, The Netherlands; 10 Institution of Clinical Sciences, Section of Rheumatology, Lund University, Lund, Sweden; 11 Skåne University Hospital, Lund, Sweden; 12 Happy Ageing Alliance, Rome, Italy; 13 University of Geneva, Geneva, Switzerland; 14 Rheumatology Unit, San Pietro Fatebenefratelli Hospital, Rome, 00189 Italy; 15 Department of Geriatric Care, Neurology and Rehabilitation, E. O. Galliera Hospitals, Genoa, Italy; 16 Department of Interdisciplinary Medicine, University of Bari “Aldo Moro”, Bari, Italy; 17 Institute for Biomedicine of Aging, Friedrich-Alexander University Erlangen-Nürnberg, Solna, Sweden; 18 County Hospital Winterthur, Winterthur, Sweden; 19 Deutsches Rheuma-Forschungszentrum Berlin, Berlin, Germany; 20 Charité - Universitätsmedizin Berlin, Berlin, Germany; 21 Saint Camillus International University of Health Sciences, Rome, Italy; 22 Department of Neurobiology, Aging Research Center, Care Sciences and Society, Karolinska Institutet and Stockholm University, Solna, Sweden; 23 Stockholm Gerontology Research Center, Stockholm, Sweden; 24 National Research Council, Neuroscience Institute, Aging Branch, Padova, Italy; 25 Faculty of Medicine and Geneva University Hospitals, Geneva, Switzerland

**Keywords:** Musculoskeletal disease, Infection, Vaccination, Older adults

## Abstract

Musculoskeletal disorders are a significant public health burden concern, projected to increase in the coming decades, and will substantially contribute to the rising prevalence of functional impairment, frailty and disability in a growing global population. Since persons with musculoskeletal disorders tend to have immune dysfunction, inflammation or be taking immunosuppressive medication, prevention of vaccine-preventable diseases (VPDs) in this group is particularly important. The European Interdisciplinary Council for Aging (EICA) and the European Society for Clinical and Economic Aspects of Osteoporosis, Osteoarthritis and Musculoskeletal Diseases (ESCEO) jointly convened a 2-day in-person and virtual meeting on 26–27 September 2023, to review the state of the evidence on the link between musculoskeletal diseases, infections and vaccines. We present here the Executive Summary of the proceedings of this meeting. We review the importance of physical activity in preventing or mitigating both musculoskeletal diseases and risk of infection. We summarize current knowledge of the impact of common VPDs on the development and progression of musculoskeletal diseases, and the role of selected vaccines in preventing onset and worsening of frailty and disability in these individuals. This report summarizes the evidence presented at the two-day meeting, highlighting the need to raise awareness among scientists, healthcare professionals, decision-makers, civil society and the general public about the long-term sequelae of VPDs, with focus on the health status of older patients with musculoskeletal diseases.

## Introduction

According to the latest estimates from the Global Burden of Disease study in 2019, approximately 1.71 billion people worldwide are living with musculoskeletal disorders, corresponding to an age-standardised rate of 210 per 1,000 persons [1], which represents a 62% increase since 1990, with the upward trend expected to continue in parallel to rapid global population aging. The rising rates of musculoskeletal disease account for a large proportion of the number of people experiencing declines in functional capacity, or living with disability, which raises the perspective of considerable burden on health and social care systems. Persons with musculoskeletal disorders may have immune dysfunction, or be taking immunosuppressive medication, or may also suffer from chronic autoimmune and inflammatory diseases. Data from the literature suggest that these conditions are associated with a higher risk of certain vaccine-preventable diseases, such as influenza or pneumococcal infections. Therefore, there is a strong rationale to review the state of the evidence regarding the link between infections and musculoskeletal diseases, and the possible role of vaccination to prevent further functional deterioration that can be associated with these conditions. The European Interdisciplinary Council for Aging (EICA) and the European Society for Clinical and Economic Aspects of Osteoporosis, Osteoarthritis and Musculoskeletal Diseases (ESCEO) jointly convened a 2-day in person and virtual meeting on 26–27 September 2023, to review the state of the evidence on the link between musculoskeletal diseases, infection and vaccines. We present here the Executive Summary of the proceedings of this meeting. Speakers were tasked with summarizing the current state of knowledge on each specific topic, in particular identifying gaps in knowledge that could be avenues for future collaborative research.

First, we review the importance of physical activity in preventing or mitigating both musculoskeletal disease and risk of infection, and how healthy ageing overall is modulated by the interplay between immunity, functional capacity, frailty and infection. Then, we summarize current knowledge of the impact of common vaccine-preventable diseases (VPDs) on the development and progression of musculoskeletal diseases, and the role of selected vaccines in preventing onset and worsening of frailty and disability in individuals with musculoskeletal diseases. Finally, we identify future activities aimed at raising awareness among scientists, healthcare professionals, decision-makers, nongovernmental organisations, civil society and the general public about the long-term sequelae of VPDs on the health and functional status of older persons with musculoskeletal diseases.

## Physical activity, infectious diseases and vaccinations

It is well established that physical activity promotes both physical and psychological well-being [2]. Indeed, exercise has a physiologically important effect on the body. During exercise, there is an increase in cardiac output, blood flow and release of stress hormones. In response to exercise, there is acute mobilization of immune cells, both during the effort and the recovery phase [3, 4]. Over time, training induces a chronic adaptation to exercise that is associated with improved immune function, increased antibody production, increased response to vaccination and even perhaps increased surveillance of cancer cells. Studies have shown that the risk of community-acquired infection, and of infectious disease-related mortality is lower in those who regularly exercise [5]. There is clear evidence that exercise provides protection against infection and improves the response to vaccination. This raises the question of whether the transcriptomic signatures of exercise in immune cells suggest a beneficial effect on immune function, and whether exercise elicits different transcriptomic immune response in young and older individuals. If exercise reduces inflammation, and enhances antioxidant defences, then it could potentially counteract the effects of aging on immune function. Evidence strongly suggests individuals who exercise regularly have better immune function, reflected in the gene expression, as well as enhanced response to vaccination. Identifying interventions that can change the aging gene expression and confer on immune cells benefits from physical activity, is currently the focus of an expanding line of research in animal models. The results of these studies may be extremely important for human health.

## Fostering healthy aging: the interdependency of infections, immunity and frailty

Frailty is undoubtedly the most important barrier to achieving healthy ageing. Definitions of frailty abound, but the most commonly used definitions are based on the phenotypical approach, i.e. physical frailty, and the deficit accumulation model [6]. Recently, the concept of multidimensional frailty has emerged based on the notion of mutual interaction among physical, functional, psycho-social and biological factors, including immunity, and strongly related to the prognosis of the older subject [7–9]. Multidimensional frailty is best captured by the Comprehensive Geriatric Assessment (CGA) and its derived tools such as the Multidimensional Prognostic Index (MPI) [10]. Indeed, the multidimensional approach based on the Comprehensive Geriatric Assessment (CGA) may not only address frailty but also enable identification of the elements for the most appropriate therapeutic strategy to be implemented in older patients with infectious diseases [11, 12]. Regardless of the conceptual framework used, frailty is a dynamic phenomenon characterised by various trajectories and transitions. Changes in frailty status mostly involve progression to a more frail state, although this is not universal [6]. Frailty has been shown to be a negative prognostic condition for infection [13] and contributes to increased mortality in adults with infectious diseases [14, 15]. Frail older people with multimorbidity have an increased risk of infection, and once infected, are also at higher risk of adverse outcomes.

Because of the epidemiological evidence of a strong association between infection and frailty, a better understanding of pathophysiology that links immunization and frailty is of paramount importance. With the growing numbers of both older individuals and those with chronic medical conditions, there is a compelling need to understand the impact frailty may have on the response to vaccination. To date, the evidence, while sparse, does show that the response to pneumococcal vaccination is dampened in frail older persons [13]. Preventing a significant loss of functional capacity and then frailty could be reliant on stronger evidence on the intrinsic value of influenza, pneumococcal disease and herpes zoster vaccination across the life-course, including older individuals and those with chronic medical conditions.

## The role of vaccination in a multidimensional approach to Sarcopenia

There is strong interdependency between infection, immunity and frailty. Future studies are needed to investigate whether there is a clear association between immunosenescence, infection and frailty, and whether enhancing the immune response in older age by vaccination could help to reduce the impact of frailty and late-life infection (often related to frailty). Clearly, infection and frailty have pathophysiological paths that intersect and intertwine throughout the lifetime. New evidence suggests targeting the neuro-immuno-endocrine system, to act on immunosenescence, inflammaging and perhaps ultimately, frailty and sarcopenia [10].

## Impact of infection on patients with musculoskeletal diseases

### Impact of COVID-19 pandemic on patients with rheumatic diseases

At the beginning of the COVID pandemic, a national registry of patients with autoimmune inflammatory rheumatic diseases (AIIRD) and diagnosed with COVID-19 was established in Israel, based on voluntary reporting by treating rheumatologists, covering 80% of the total population of Israel. This registry provided insights into the profiles of AIIRD patients across the consecutive waves of the COVID pandemic, both before and after vaccines became available [16]. Reports from this registry indicate that, despite similar demographic and clinical characteristics, a smaller proportion of AIIRD patients had negative outcomes in the 4th COVID wave, as compared with the first 3 waves, in terms of disease severity, hospitalization and death. Furthermore, COVID-19 infection did not appear to influence AIIRD activity in the 3 months post-recovery [16]. In another study including 686 patients with AIIRD who were compared to 121 individuals from the general population, it was reported that AIIRD patients responded well to vaccination, with a seropositivity rate of 86% [17]. The factors associated with reduced immunogenicity were older age and treatment with glucocorticoids, rituximab, Mycophenolate mofetil (MMF), and abatacept [17]. A review of the impact of disease modifying anti-rheumatic drugs (DMARDs) on vaccine immunogenicity in patients with inflammatory rheumatic and musculoskeletal diseases similarly reported evidence that rituximab has a clinically meaningful impact on response to influenza, pneumococcal and SARS-CoV-2 vaccines [18].

In the earliest trials of COVID-19 vaccines, patients with AIIRD were generally excluded [19], and thus, there was a relative scarcity of data pertaining to the potential association between COVID-19 vaccination and the possibility of autoantibody development and/or disease flares. It has since been shown in several studies that very low numbers of patients develop autoantibodies, and even fewer develop clinical autoimmunity, and existing evidence does not support an association between vaccination and disease flares [20–22]. Nevertheless, there is clear evidence that rituximab-treated patients with AIIRD do not mount an adequate humoral response after COVID-19 vaccination, even with boosters.

Overall, the different drugs in the treatment for AIIRD may impact the patient’s immunogenicity after receipt of vaccines against COVID-19 and other diseases. In the specific population of patients with rheumatic diseases, this needs to be taken into account to ensure that adequate prevention is provided against common vaccine-preventable diseases and their complications.

### Prevalence and risk factors for herpes zoster in rheumatoid arthritis

Herpes zoster is caused by reactivation of the Varicella Zoster Virus (VZV). Shingles has a characteristic presentation, with a painful vesicular rash along a dermatome. The main risk factors for shingles include older age, female sex, a positive family history, immunosuppression (e.g. HIV or cancer), and also comorbidities such as Systemic Lupus Erythematosus (SLE), Rheumatoid Arthritis (RA) or Inflammatory Bowel Disorder (IBD), amongst others [23]. Indeed, there is an increased incidence of herpes zoster in individuals with rheumatic diseases compared to the general population. It has been reported that among patients with RA, those with greater disease severity are at higher risk of herpes zoster [24], and also that the risk may be higher among patients taking certain drugs for the treatment of their RA [24–26]. Taken together, these findings underline the importance of providing vaccination against herpes zoster as early as possible for patients with RA to prevent further complications and costs. Local differences in reimbursement policies for herpes zoster vaccination may hamper efforts to ensure widespread vaccination among patients with rheumatic diseases.

### Impact of influenza vaccination on the association between hip fracture and seasonality

The majority of hip fractures are caused by either a fall or trauma, often associated with bone fragility because of osteoporosis (i.e. low bone mineral density). Hip fracture may have serious functional repercussions, especially for older adults. Up to 50% of older adults who could walk before a hip fracture never recover the same level of walking capacity or functional ability after the event [27]. Epidemiological studies have reported that there are seasonal variations in the frequency of falls, with higher rates observed in the winter months, due to reduced physical activity, winter bone loss [28] or environmental conditions associated with weather conditions (including icy conditions conducive to slipping) [29]. Other factors may also be implicated, such as outside temperature [30]. The possible mechanisms explaining this finding include decreased dexterity, neuromuscular function and variations in blood pressure during low temperatures. Reduced physical activity during poor weather could also promote or worsen osteoporosis, putting these patients at increased risk. Another possible mediator of the seasonal patterns in hip fracture could be vitamin D, as there are naturally lower vitamin D levels during seasons with lower sun exposure [31, 32].

There is a rationale to argue that influenza vaccination may be a mediator in the seasonal pattern of hip fracture. In a retrospective cohort of nursing home residents in the United States, hospitalization for influenza-like illness (ILI) was associated with an average 13% increase in the risk of hip fracture hospitalization [33]. This relationship may be bi-directional, whereby older individuals who are hospitalized for hip fracture may be more likely to receive influenza vaccination, or those who are in contact with healthcare services to obtain vaccination may simultaneously receive treatment for osteoporosis. Pending robust data from large, epidemiological studies, no firm conclusions can be drawn regarding the direct influence of influenza vaccination on the risk of hip fracture. However, there is evidence to suggest that the risk of ILI and hip fracture are positively correlated, and therefore, preventing ILI and influenza infection by means of vaccination could likely moderate this relationship.

### Risk of serious pneumococcal infections up to 10 years after a dose of pneumococcal conjugate vaccine in patients with established rheumatic disease

Pneumococcal vaccination is recommended for patients with immunosuppression due to treatment for AIIRD by the US Advisory Committee on Immunization Practices (ACIP) [34], the European League Against Rheumatism (EULAR) [35] and national authorities in numerous countries. However, studies investigating the clinical efficacy of pneumococcal vaccination among patients with AIIRD are quite scarce [36]. Nagel et al. investigated putative serious pneumococcal infection rates up to 10 years before and after administration of pneumococcal 7-valent conjugate vaccine (PCV7) in patients with inflammatory arthritis, compared to unvaccinated arthritis patients [37]. There was a significant, 53% reduction in the relative risk of serious infection after vaccination with PCV7. Similarly, a 46% relative risk reduction was observed for all serious pneumococcal infections in vaccinated patients. This study shows that there was a significant relative risk reduction for pneumonia and all serious pneumococcal infection up to 10 years after a single dose of PCV7 in both RA and Spondyloarthritis patients receiving different anti-rheumatic treatments. These findings underline the potential benefit from vaccination against pneumococcal disease in patients with AIIRD.

## Vaccine recommendations for adults suffering from immune-mediated inflammatory diseases

Patients with AIIRD, such as RA and Systemic Lupus Erythematosus (SLE), are at higher risk for serious infections, due to disease-related immune dysfunction and immunosuppressive medication. Because of this risk, vaccination is an important part of care. Vaccination against influenza, COVID-19, and *Streptococcus pneumoniae* is recommended for patients with AIIRD by most major national and international medical societies, including the American College of Rheumatology (ACR) and the European Alliance of Associations for Rheumatology (EULAR) [35, 38]. Non-live vaccines should be given to patients with AIIRD in accordance with indications. For example, pneumococcal and COVID-19 vaccination are recommended for all patients with AIIRD, who are either already receiving, or planning to receive immunosuppressive therapy. Annual influenza vaccination is also recommended unless contraindicated [39]. Of note, and in line with the low level of expert consensus on this point, caution should be exercised with live attenuated vaccines in patients with AIIRD. Vaccination should ideally be completed ≥ 2 weeks prior to the start of immunosuppression to achieve maximal immune response. When vaccination cannot be given prior to initiation of immunosuppressive therapy, the vaccine should be given as soon as possible after the start of treatment, and ideally during a period when immunosuppression is low.

The different immunosuppressive therapies used in AIIRD may have differential effects on vaccine immunogenicity. Nevertheless, although certain agents may mitigate the immune response, vaccination is still expected to yield a protective benefit in the majority of patients [40–43].

Regarding the timing of vaccination, all vaccines should ideally be administered during the window of opportunity prior to initiation of immunosuppressive therapy. However, in practice this can be challenging, and therefore, any vaccines that need to be given after the start of immunosuppressive agents should be given as soon as possible, and during the quiescent phase of the disease where possible. Live vaccines should be given at least 2 weeks before the start of any immunosuppressive therapy, and at least 4 weeks before the start of more potent immunosuppressive agents.

In summary, patients with AIIRD are at increased risk of serious infections due to both immune dysfunction and use of immunosuppressive medication, and therefore, administration of routine vaccinations is a cornerstone of care for these patients. Patients with AIIRD should be up-to-date with all recommended vaccinations that are appropriate for their age and risk group, mostly notably seasonal influenza, COVID-19, herpes zoster and pneumococcal vaccines. In particular, there is a compelling need to raise awareness among healthcare professionals about the need for, and benefit of vaccination in AIIRD patients.

## Vaccine development and decision-making

The basic requirements for all vaccines are that they be safe, of good quality, and effective. They must provide a high level of protection at a public health level, have few side effects, and have a reasonable cost. The development process for vaccines is usually very long, and ranges from early non-clinical testing in vitro and in vivo, through the three phases of clinical testing, to check the vaccine is safe and effective, before moving to regulatory approval, reimbursement/coverage decisions and then post-marketing pharmacovigilance and monitoring. In the approval of new medicines, including vaccines, marketing authorization is given after the drug passes all the phases of clinical testing, and proof of its efficacy, safety and quality. Then, there is a national value assessment, where National Immunization Technical Advisory Groups (NITAGs) or other bodies will advise the local ministry for health (usually the national-level decision-making), with a view to (sub)national implementation, at a local level.

It is the role of national authorities to adopt a new vaccine into their national health system, and there are significant differences between countries regarding who is responsible for this process. Stakeholders in vaccine decision-making include the Strategic Advisory Group of Experts on Immunization (SAGE), (which advises the World Health Organization (WHO) on overall global policies and strategies, ranging from vaccines and technology, research and development, to delivery of immunization and its linkages with other health interventions), and the Regional Technical Advisory Groups on Immunization (RITAG).

There are a number of key issues to be considered when deciding on the introduction of a vaccine, relating to the disease, the vaccine, the strength of the immunization programmes, the cost effectiveness and the healthcare system overall.

In recent years, the broader economic impacts of vaccines have garnered increasing attention. Indeed, vaccines not only have individual health-related impacts, but also impact on wider aspects of society, including productivity; community and system level issues, with broader macroeconomic repercussions (e.g. less frailty, more stable workforce) [44]. These kinds of considerations, while not comprehensive or country-specific, may be particularly substantial in the case of a pandemic such as that caused by SAR-CoV-2, which affected working-age adults. In such contexts, the multidisciplinary composition of NITAGs is also an advantage for the adoption of vaccination recommendations when there is little, if any scientific evidence, and expert opinions from several disciplines are needed to reach a balanced consensus. Future perspectives for improvement in this area could include the adoption of a “life-course” approach to vaccination by NITAGs, whereby each vaccine would no longer be considered in isolation, but rather, integration of life-course vaccination would be promoted as a pathway to healthy aging.

## Conclusion

Musculoskeletal disorders represent an unprecedented public health burden, which will grow in the coming decades. This global trend has wide-ranging consequence on individuals, population health and health systems and the broader social and economic landscape. Persons with musculoskeletal disorders may have immune dysfunction, inflammation or be taking immunosuppressive medication, putting them at higher risk of certain VPDs. The state of evidence on the link between musculoskeletal diseases, infection and vaccines reviewed in the 2-day meeting organized by the EICA and ESCEO, reveals numerous key points: first, the importance of physical activity in preventing or mitigating both musculoskeletal disease and infection; second, the impact of VPDs on the development and progression of musculoskeletal diseases, and the role of selected vaccines in preventing onset and worsening of frailty and disability in patients with musculoskeletal diseases; and third, the importance of raising awareness among rheumatologists and other physicians of the need for vaccination against common VPDs for patients suffering from musculoskeletal disorders. For all topics presented and discussed during the meeting, we found that there is scarce evidence regarding older persons and research in this specific population is strongly needed.

## Data Availability

No datasets were generated or analysed during the current study.
